# Interaction between Graphene-Based Materials and Small Ag, Cu, and CuO Clusters: A Molecular Dynamics Study

**DOI:** 10.3390/nano11061378

**Published:** 2021-05-23

**Authors:** Isabel Lado-Touriño, Alicia Páez-Pavón

**Affiliations:** School of Architecture, Engineering and Design, Universidad Europea de Madrid, 28670 Villaviciosa de Odón, Spain; ALICIA.PAEZ@universidadeuropea.es

**Keywords:** antibacterial activity, nanoclusters, molecular dynamics, graphene-based materials, polyethylene glycol

## Abstract

The excessive use of antibiotics has contributed to the rise in antibiotic-resistant bacteria, and thus, new antibacterial compounds must be developed. Composite materials based on graphene and its derivatives doped with metallic and metallic oxide nanoparticles, particularly Ag, Cu, and Cu oxides, hold great promise. These materials are often modified with polyethylene glycol (PEG) to improve their pharmacokinetic behavior and their solubility in biological media. In this work, we performed molecular dynamics (MD) simulations to study the interaction between small Ag, Cu, and CuO clusters and several graphene-based materials. These materials include pristine graphene (PG) and pristine graphene nanoplatelets (PGN) as well as PEGylated graphene oxide (GO_PEG) and PEGylated graphene oxide nanoplatelets (GO-PEG_N). We calculated the adsorption energies, mean equilibrium distances between the nanoparticles and graphene surfaces, and mean square displacement (MSD) of the nanoclusters. The results show that PEGylation favors the adsorption of the clusters on the graphene surfaces, causing an increase in adsorption energies and a decrease in both distances and MSD values. The strengthening of the interaction could be crucial to obtain effective antibacterial compounds.

## 1. Introduction

The excessive use of antibiotics has contributed to the rise in antibiotic-resistant bacteria, and new antibacterial compounds must be developed. Graphene is a 2D monolayer material with long edges and large accessible surfaces, which give it an excellent ability to immobilize diverse molecules including nanoparticles or drugs. Hu et al. [1] first reported the antibacterial activity of graphene oxide (GO) and reduced graphene oxide (rGO) nanosheets against *E. coli* bacteria. Many subsequent studies showed that pristine graphene, GO, and rGO have all antibacterial activity [2,3,4,5,6,7,8,9]. As graphene-based materials tend to aggregate due to strong van der Waals interactions, thus reducing their effective surface and antibacterial activity, they are modified with metals, oxides, polymers, or a combination of these [10,11,12,13,14,15,16]. In particular, the use of Ag, Cu, and their oxides adsorbed on the graphene surface appears promising [17,18,19,20,21,22,23,24,25]. Moreover, graphene-based composites, prepared by incorporating both nanoparticles and polymers, exhibit synergistic antibacterial effects [16,26]. The polymers used include polyethylene glycol (PEG), which is a polymer that helps to obtain stable graphene−PEG materials dispersible in most biocompatible solvents [27,28]. For instance, PEG-GO remains highly dispersed in serum solutions [29] and shows good pharmacokinetic behavior [30] and biocompatibility [31,32].

Molecular dynamics (MD) simulations allow the study of materials from a molecular viewpoint, providing information on an atomic scale usually inaccessible to experimental techniques [33]. The interaction between graphene-based materials and different nanoparticles and polymers has been described in many research works by MD [34,35,36,37,38,39,40,41,42]. Several other authors have performed molecular modeling studies on the interaction between graphene and PEG [43,44,45,46,47,48].

In this work, we carried out MD simulations of the adsorption of small Ag, Cu, and CuO clusters on pristine graphene monolayers (PG), PG nanoplatelets (PGN), PEGylated graphene oxide monolayers (GO_PEG), and PEGylated graphene oxide nanoplatelets (GO-PEG_N). The work focuses on a comparison of the behavior of these three nanoclusters interacting with the above-mentioned graphene-based materials and allows completing our previous results on Ag and Cu nanoclusters interacting with a graphene surface [35]. In our new simulations, much longer simulation lengths are used, and more systems have been included in the calculations: PGN, GO-PEG_N, and CuO clusters. We calculated adsorption energies, mean equilibrium distances between the nanoparticles and graphene surfaces, and mean square displacement (MSD) of the nanoclusters. The results show that PEGylation favors the adsorption of the clusters on the graphene surfaces, causing an increase in adsorption energies and a decrease in both distances and MSD values. Thus, this work can provide important clues on the way that graphene-based materials can be modified to become effective antibacterial compounds.

## 2. Models and Calculation Method

MD simulations were done with the Forcite module of the Materials Studio 9 software [49] in the NVT ensemble (constant number of particles N, constant volume V, and constant temperature T) at 298 K during 10 ns of simulation length. The interactions between atoms were calculated using the COMPASS forcefield, which is a force field parameterized using experimental and ab initio results that enables an accurate prediction of various gas-phase and condensed-phase properties of several organic and inorganic materials [50], including graphene and graphene-based materials [44,51,52]. The temperature was kept constant using a Nosé-Hoover thermostat^.^ [53,54]. Electrostatic and van der Waals interactions were calculated using an atom-based summation method with a cut-off of 12.5 Å.

**Figure 1:**
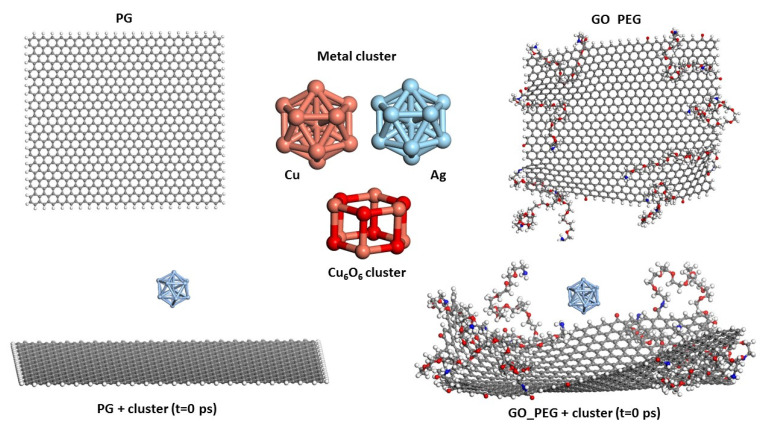
Models of PG, GO_PEG, and clusters. The starting structures used in the simulations before cluster adsorption on both surfaces are shown in the lower part of the figure.

**Figure 2:**
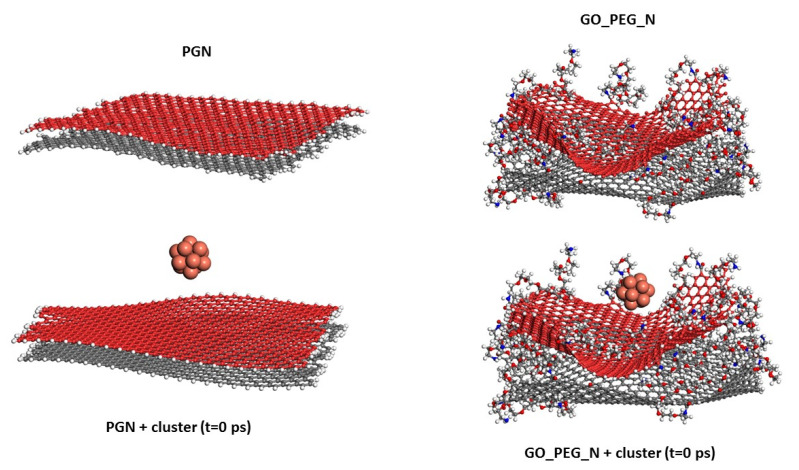
Models of PGN and GO_PEG_N. The starting structures used in the simulations before Cu cluster adsorption on both surfaces are shown in the lower part of the figure. Each basal graphene plane in the nanoplatelets is displayed in a different color for a clearer view.

The starting configurations used for the production trajectories are shown in Figure 1 (graphene monolayers) and Figure 2 (nanoplatelets). A basal graphene sheet with a width of 46.5 Å and a length of 56.2 Å was used for all structures. To build these starting structures, all the systems were subjected to an annealing procedure composed of NVT MD simulations and further steepest descent and conjugate gradient energy minimization cycles at each temperature. This annealing procedure allows exploring the conformational space for low-energy structures, which will be used as starting configurations, by periodically increasing and then decreasing the temperature (from 300 up to 1000 K) of a classical dynamics trajectory, to avoid trapping the structure in local energy minima. For the sake of comparison, PG, PGN, GO_PEG, and GO-PEG_N were constructed. The PEGylated structures were functionalized with eight short chains of PEG (degree of polymerization *n* = 10). In the interest of saving computational time, only two layers of graphene were used to model the nanoplatelets. In the PEGylated nanoplatelets, both layers had the same grafting density, i.e., the number of PEG chains attached to the edges of the individual GO layers. In these starting structures, the mean final distances between the basal planes of graphene were 3.7 and 6.9 Å for PGN and GO-PEG_N, respectively. The clusters used to represent the metal nanoparticles were composed of thirteen atoms arranged in an icosahedral shape. This number was chosen because of the well-known stability of this regular icosahedron geometry for Cu and Ag nanoclusters [55,56]. For CuO nanoparticles, a Cu_6_O_6_ cluster consisting of two hexagonal layers, one above the other, was chosen. This structure was found to be stable by a density functional theory calculation [57]. No constraints were imposed on the systems, so that all molecules could move freely over the whole simulation length. At the beginning of the calculation, the clusters were placed far from the surfaces, at distances of about 8 Å, as can be seen in the lower part of both Figures, and they were left to evolve with time.

The adsorption energy of the clusters on the surface was calculated from the following equation:

E = E_GBM+cluster_ − (E_GBM_ + E_cluster_) (1)

where E_GBM+cluster_ is the mean equilibrium potential energy of the graphene-based material (GBM = graphene-based materials = monolayers or nanoplatelets) interacting with the cluster, and E_GBM_ and E_cluster_ are the mean equilibrium potential energies of the isolated graphene-based materials and clusters, respectively (all the energies were calculated as average values from three independent 10 ns simulations. In turn, the result of each individual simulation is the average of the energy values obtained during the last 5 ns of simulation). As the energy values are negative, the more negative the adsorption energy is, the stronger the interaction. Previous studies have shown that this adsorption energy could be a good estimate of the binding strengths of adsorbate–adsorbent systems [58].

The mean square displacement (MSD) of the clusters, which measures the spatial extent of clusters random motion, was calculated from their positions over simulation length according to:

MSD = Σ < (r_i_(0) − r_i_(t))^2^ > /N (2)

where r_i_(0) is the reference position of the cluster, r_i_(t) is its position at time t, and N is the number of atoms of the cluster. Thus, MSD was calculated from the particle positions obtained from the trajectory using a time step of 5 ps.

MSD can be related to time through the following equation:

MSD = K_α_t^α^ (3)

where K_α_ is the generalized diffusion coefficient and α is the diffusion exponent [59,60]. The value of this exponent is related to the mechanism of diffusion.

## 3. Results and Discussion

### 3.1. Adsorption Energies

The adsorption energy values calculated from Equation (1) are presented in Table 1.

From the values listed in Table 1, it can be concluded that the strength of the interaction depends on both the chemical nature of the cluster and the functionalization of the graphene-based material. All the clusters interact more weakly with pristine materials than with functionalized materials in both monolayers and nanoplatelets. The Cu_6_O_6_ cluster is the most attracted by the surface and the Cu_13_ cluster is the least attracted. Ag_13_ lies in between. The introduction of PEG chains favors the adsorption process, increasing the adsorption energy. In addition, the nanoplatelets seem to increase the adsorption energy in all cases. This last trend should be confirmed by increasing the number of layers of the nanoplatelets in future studies.

Here, we must note the qualitative nature of these results. It is well known that cluster geometry and size influence their adsorption energy and reactivity on graphene surfaces, and several authors have shown that quantum mechanical calculations are needed to fully understand and quantify this kind of interaction [61]. Even when using density functional theory calculations, the results vary widely (adsorption energies ranging from a few kcal/mol to a hundred kcal/mol and cluster–graphene surface distances ranging from 2 to 3 Å) depending on the molecular models, the calculation method, or the number of atoms included in the nanocluster [62,63,64]. What is clear is that metal d-orbitals are involved in charge transfer processes between the clusters and the graphene surface, although this charge transfer is weak in some cases, and the bonding can be mainly attributed to van der Waals forces. Cu and Ag are examples of that, as it has been demonstrated by numerous calculations [65,66]. On the other hand, experimental results are diverse and show that the structure of the material after the interaction depends on many factors, including the initial graphene form and the methods used for its synthesis [67]. In addition, due to limited computational resources, our simulations were performed in vacuum, and we are aware that this may influence the results. Thus, the small clusters and the graphene model used in this work represent a first step to compare qualitatively the three types of particles, and we realize that they cannot capture the whole complexity of real systems.

Cu_6_O_6_ interacts strongly with both pristine and functionalized surfaces, although the adsorption process is improved by the presence of PEG chains, as already observed for Cu and Ag clusters. We think the atomic charges of the cluster increase the electrostatic attraction between the cluster and surface, although the exact nature of the interaction should be verified by more accurate quantum mechanical calculations. Sun et al. [68] determined that during the process of nucleation of graphene on Cu-based substrates, the adsorption energies of C were larger for copper oxides than for metallic Cu. Ko et al. [69] prepared CuO nanoparticles covered with a monolayer graphene shell, in which strong C-O-Cu links were formed. Using density functional theory calculations, Mohammadi-Manesh et al. [70] calculated the binding energy of different configurations of adsorption of Cu and CuO on graphene surfaces and found that the interaction between Cu and graphene is physical and the interaction between CuO and graphene is chemical.

Even if the calculation method used in this work cannot describe the formation of chemical bonds between the cluster and the graphene surfaces, the results obtained agree with those obtained from more accurate quantum chemical calculations.

### 3.2. Cluster–Surface Distances

The mean equilibrium distances between the cluster and nearest surface atoms are shown in Table 2. The reduction of the distances used in the starting structures (8 Å) indicates that the clusters approached the graphene surface as the simulation progressed and attained an equilibrium value toward the end of the calculation. The values listed in Table 2 show that the mean distance between the clusters and the pristine surfaces is about 2.9 Å for both monolayers and nanoplatelets. The introduction of PEG chains brings the cluster closer to the graphene surface leading to reduced distances of about 2.4–2.5 Å. Cluster–pristine material distances are larger than cluster–functionalized material distances, which correlates well with improved adsorption energies for GO_PEG-containing materials.

The distances shown in Table 2 for the pristine systems are similar to those found in previous quantum mechanical [65,66] and MD [71,72] simulations of the interaction between graphene and Cu and Ag. DFT results give distances of 2.3–3.9 and 2.8 Å for Cu and Ag, respectively. The distance found by MD between Ag and graphene is 3 Å. MD results for Cu show a range of binding distances varying between 2 and 3.4 Å, depending on the orientation of Cu relative to graphene. They are related to weak bonding (physical adsorption) attributed to the full occupancy of metal d orbitals [73,74].

**Figure 3:**
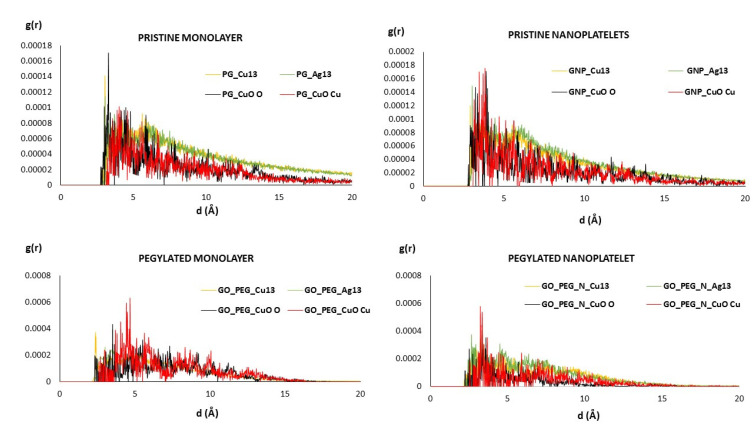
RDF of metallic clusters and graphene-based surfaces. For the PEGylated structures (bottom), only the RDF of the clusters and the nearest chains of PEG are shown.

The radial distribution functions (RDF) of clusters and graphene-based surfaces are shown in Figure 3. These plots give us an idea of the spread of distances between the clusters and surfaces. All plots present maxima between 2.5 and 5–6 Å, and there are no large differences between the different models. It must be noted that the mean equilibrium distances shown in Table 2 represent distances between the cluster and nearest surface atoms, which must not necessarily coincide with maxima in Figure 3. In the case of the Cu_6_O_6_ cluster, the plots corresponding to the Cu atom (shown in red) are slight shifted to larger values, which is in accordance with the results shown in Table 2. Figure 3 also shows that in the PEGylated systems, distances are somewhat smaller.

**Figure 4:**
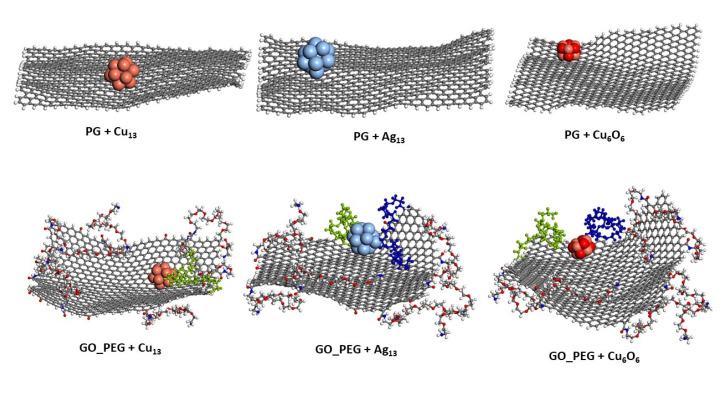
Representative position of the nanoclusters for pristine and functionalized graphene monolayers after 10 ns of simulation length. The PEG chains closest to the nanoparticles are shown in blue and green.

**Figure 5:**
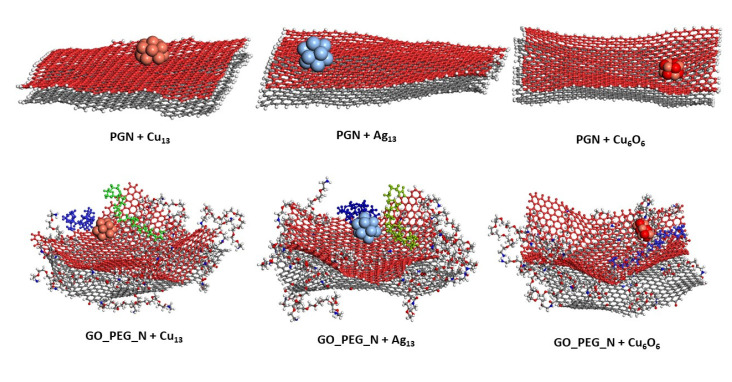
Representative position of the nanoclusters for pristine and functionalized graphene nanoplatelets after 10 ns of simulation length. The PEG chains closest to the nanoparticles are shown in blue and green.

Figure 4 and Figure 5 show the representative positions of the clusters on the monolayer surface (Figure 4) and the nanoplatelet surface (Figure 5). Only the result obtained from one of the simulations is depicted here. In the case of the GO_PEG surfaces, it was observed that the clusters could stick to any lateral PEG chain of the layers, and once stuck, they remained bonded to them at a distance of about 2.5–2.6 Å in all cases (see Table 2). In the case of CuO clusters, this distance corresponds to O-PEG distances. In all cases, the Cu atom in the cluster was located at somewhat larger distances. PEG is frequently used to prepare metal and metal oxide nanoparticles in solution [75,76,77], as it is well known that PEG molecules strongly adsorb on metal nanoparticles surfaces by coordination through the ether bond of PEG and prevent their aggregation [78]. The capping capability of PEG could explain the strong attraction it exerts on the clusters and their subsequent immobilization on the graphene surfaces.

### 3.3. Mean Square Displacement

**Figure 6:**
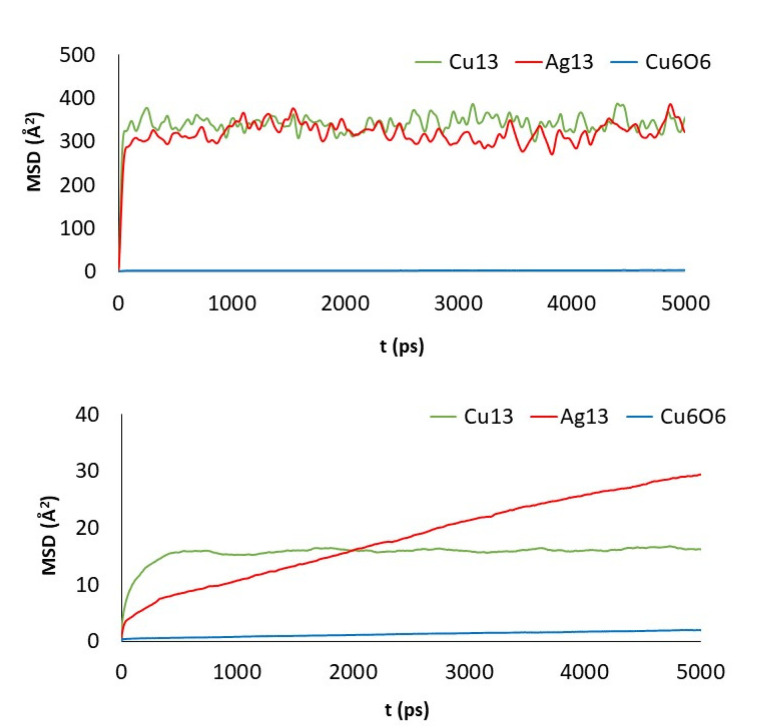
MSD of the clusters versus simulation length: PG (top) and GO_PEG (bottom).

**Figure 7:**
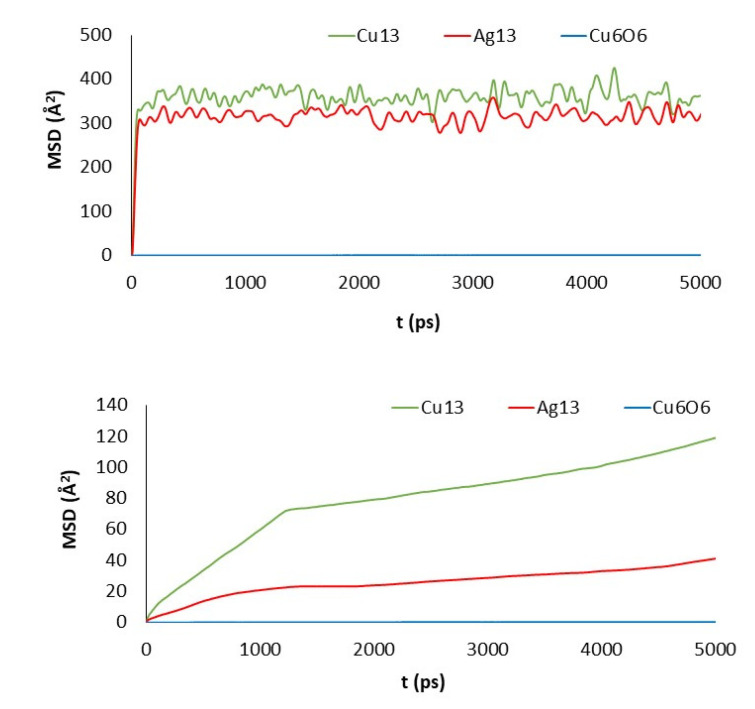
MSD of the clusters versus simulation length: PGN (top) and GO_PEG_N (bottom).

Although a detailed analysis of the mechanisms of cluster diffusion is beyond the scope of this work, we think that the MSD of the cluster center of mass can help shed more light on the differences observed in adsorption energies. Total MSD is plotted versus simulation length in Figure 6 and Figure 7 for monolayers and nanoplatelets, respectively. Larger MSD values indicate greater cluster mobility.

Cu_13_ and Ag_13_ clusters interacting with the pristine materials present the highest MSD values. Both PG and PGN weakly attract both clusters, which keep constantly moving over the material surface. However, the functionalization of the graphene layers drastically reduces their mobility (see bottom plots in Figure 6 and Figure 7). The PEG chains act by trapping the clusters and blocking their displacement over the surface. The Cu_6_O_6_ cluster shows a completely different behavior as its MSD values are low (blue line plots) in both the pristine and functionalized materials.

**Figure 8:**
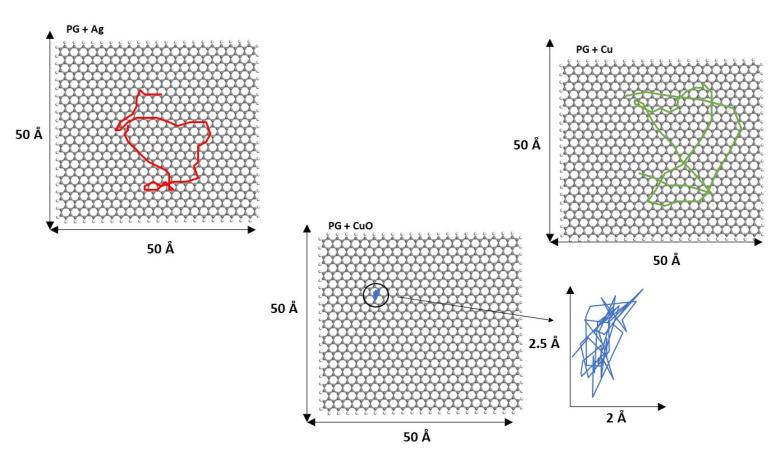
Diffusion trajectories of Ag_13_, Cu_13_, and Cu_6_O_6_ over PG during the last 250 ps of simulation length. The diffusion trajectory of the Cu_6_O_6_ cluster is magnified for a clearer view.

**Figure 9:**
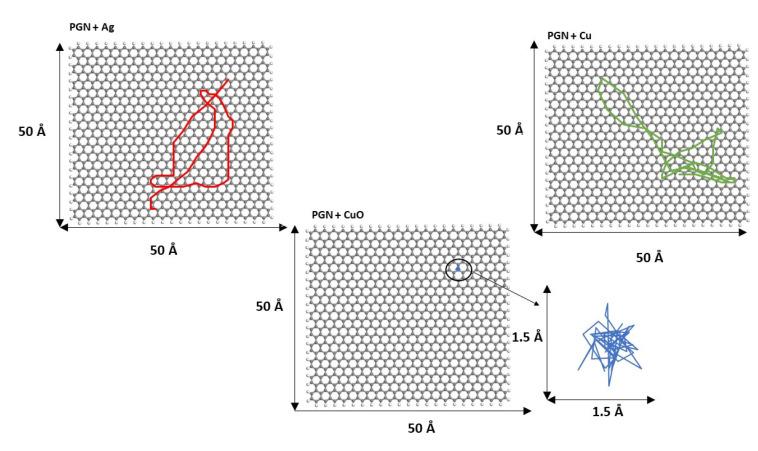
Diffusion trajectories of Ag_13_, Cu_13_, and Cu_6_O_6_ over PGN during the last 250 ps of simulation length. The diffusion trajectory of the Cu_6_O_6_ cluster is magnified for a clearer view.

To show more clearly the cluster movement, the diffusion trajectories of the cluster center of mass over the graphene surface during the last 250 ps of simulation length for both PG and PGN are displayed in Figure 8 and Figure 9, respectively. Some video representative of the different cluster dynamics is included in the Supplementary Materials (Videos S1, S2, and S3).

**Figure 10:**
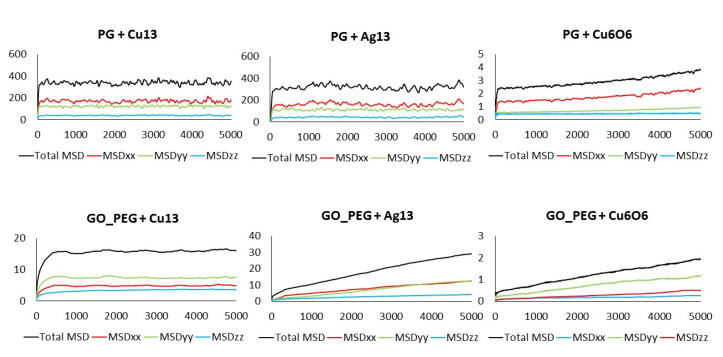
Total MSD (black) and MSD in x, y, and z directions of the clusters interacting with pristine and functionalized graphene monolayers. Axis units: time in ps (x), MSD in Å^2^ (y).

**Figure 11:**
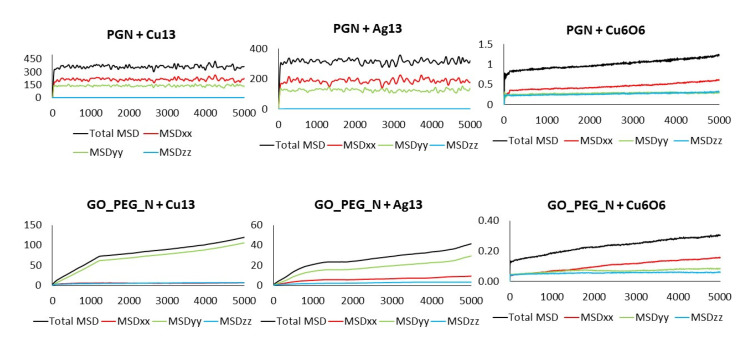
Total MSD (black) and MSD in x, y, and z directions of the clusters interacting with pristine and functionalized graphene nanoplatelets. Axis units: Time in ps (x), MSD in Å^2^ (y).

**Figure 12:**
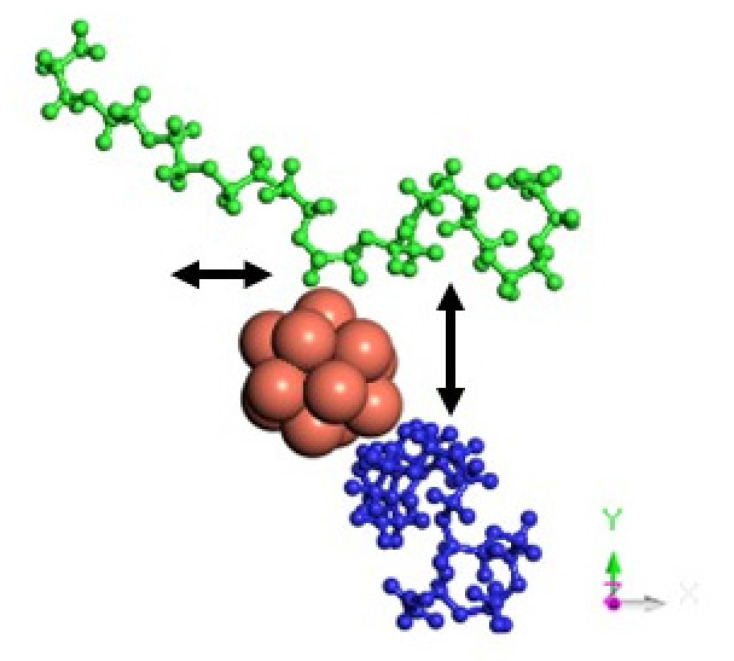
Motion in the xy plane of a Cu_13_ cluster interacting with two PEG chains of a GO_PEG surface.

The MSD values in all directions are shown in Figure 10 and Figure 11 for monolayers and nanoplatelets, respectively. The graphene surfaces are oriented parallel to the xy plane. As can be inferred from these figures, the MSD in the z direction is approximately zero (green line), as once the cluster approaches the basal plane, no vertical displacement from the surface is observed. MSD_xx_ and MSD_yy_ components have non-zero values due to the movement of the cluster over the surface and parallel to it during the whole simulation length. In the functionalized structures, the cluster may carry out slight movements between or along PEG chains (see Figure 12), although it remains bonded to them. Therefore, MSD_xx_ and MSD_yy_ present low non-zero values. An example of this dynamic behavior is included in the Supplementary Material (Video S4).

The MSD values corroborate the trends followed by the adsorption energies: the more dynamic the cluster is, the lower the adsorption energy. In agreement with our results, Gervilla et al. [79,80] studied the diffusion of Cu and Ag adatoms and clusters on graphene, using ab initio and classical molecular dynamics simulations. They found clusters diffused by a super-diffusive mechanism (α > 1 in Equation (3)), which is characterized by a continuous displacement of the clusters over the graphene surface without being trapped by adsorption sites. They attributed this behavior to a flat potential energy landscape on the surface, which facilitated the cluster diffusion. Manade et al. [74] estimated the diffusion energy barrier of Cu and Ag adatoms on graphene and arrived at the same conclusion.

Due to their higher adsorption energy, the MSD values of the Cu_6_O_6_ cluster are low on both the pristine and functionalized surfaces. Once it was attracted by the surface, it remained stuck to it (see Video S2).

## 4. Conclusions

MD simulations were carried out to study the interaction between Cu_13_, Ag_13_, and Cu_6_O_6_ clusters on pristine and functionalized graphene-based materials with the goal of finding the best structures for adsorption. Adsorption energies, cluster–surface distances, and MSD values show that both the chemical nature of the cluster and the PEGylation of the surface are critical to strengthen the interaction between the clusters and surfaces. The introduction of PEG chains favors the adsorption of the three clusters by acting as trapping sites, thus reducing the distances between the clusters and graphene surfaces and increasing the adsorption energy. MSD values also point in this direction. Pristine materials weakly attract Cu_13_ and Ag_13_ clusters, which keep constantly moving over them. PEG chains act by blocking the displacement of the nanoparticles, which are attracted by their functional groups, thus reducing MSD values. Cu_6_O_6_ is more strongly attracted than the metal clusters by both pristine and functionalized materials, as can be inferred from adsorption energies and MSD values. The forcefield used in this work does not allow reproducing the formation of chemical bonds between atoms. Previous ab initio results on metallic clusters deposited on pristine graphene and graphene oxide surfaces show that there may be oxidation of the particles and the formation of covalent bonds, which strengthens the interaction and favors the adsorption process. To correctly describe the behavior of these systems, more accurate quantum mechanical calculations are needed. In addition, the use of an experimentally associated medium (e.g., water) would enable to address in more detail the equilibrium configurations of the structures, as well as the energetic affinity between the clusters and the graphene-based surfaces. Although these more demanding simulations allows gain a deeper insight into the mechanisms that control the interaction between graphene-based materials and nanoparticles, we think our MD results can provide useful qualitative information and allow handling a larger number of atoms in shorter computational times (typical CPU running times of 10–13 h using an Intel^®^ Xeon^®^ Quad-core (4 Core) were needed for systems containing 3389 atoms). In future work, DFT calculations will be done to validate these results and be able to consider our molecular dynamics simulations as a quicker reliable method to handle these kinds of systems. Another important aspect that should be considered is the use of a solvent in the calculations. The inclusion of water may have severe effects to several important parameters, such as the equilibrium configurations of the surfaces, the spatial arrangement and the dynamics of the metallic clusters, as well as the energetic affinity between the clusters and the surfaces. Thus, future simulations must include solvent to get a more realistic analysis of real systems.

## Figures and Tables

**Table 1:** Average ΔE values for the adsorption of Cu, Ag, and CuO nanoclusters on pristine and functionalized graphene-based materials. The values obtained from each individual simulation are shown in parentheses.

Cluster	System	E (kcal/mol)
Cu_13_	PG	−13.72 (−9.11, −14.27, −17.78)
	GNP	−18.55 (−16.65, −17.91, −21.09)
	GO_PEG	−27.69 (−22.17, −29.32, −31.58)
	GO_PEG_ N	−89.54 (−79.11, −85.76, −103.75)
Ag_13_	PG	−29.11 (−25.37, −28.83, −33.13)
	GNP	−37.53 (−30.54, −37.44, −44.61)
	GO_PEG	−64.05 (−57.89, −60.45, −73.81)
	GO_PEG_ N	−116.96 (−99.54, −109.67, −141.67)
Cu_6_O_6_	PG	−77.83 (−59.54, −82.37, −91.78)
	GNP	−114.18 (−102.54, −118.02, −121.98)
	GO_PEG	−136.01 (−127.27, −130.32, −150.44)
	GO_PEG_ N	−220.64 (−191.09, −227.43, −243.40)

**Table 2:** Mean equilibrium distances between the cluster and nearest graphene surface atoms. The values obtained from each individual simulation are shown in parentheses.

Cluster	System	d (Å)
Cu_13_	PG	2.92 (2.85, 2.90, 3.01)
	GNP	2.92 (2.84, 2.91, 3.01)
	GO_PEG	2.45 (2.37, 2.46, 2.52)
	GO_PEG_ N	2.44 (2.20, 2.46, 2.66)
Ag_13_	PG	3.01 (2.97, 3.02, 3.04)
	GNP	2.99 (2.95, 2.98, 3.04)
	GO_PEG	2.51 (2.38, 2.56. 2.59)
	GO_PEG_ N	2.44 (2.34, 2.40, 2.58)
Cu_6_O_6_	PG	2.98 (2.94, 2.99, 3.01) (O) 3.45 (3.21, 3.33. 3.45) (Cu)
	GNP	2.93 (2.78, 2.97, 3.04) (O) 3.01 (2.92, 3.07, 3.04) (Cu)
	GO_PEG	2.55 (2.34, 2.52, 2.79) (O) 3.11 (2.94, 3.07, 3.32) (Cu)
	GO_PEG_ N	2.63 (2.54, 2.61, 2.74) (O) 2.94 (2.72, 2.99, 3.11) (Cu)

## Data Availability

No new data were created or analyzed in this study. Data sharing is not applicable to this article.

## Supplementary Materials

The following are available online at https://www.mdpi.com/article/10.3390/nano11061378/s1, Video S1: Cu diffusion on a pristine graphene surface, Video S2: CuO diffusion on a pristine nanoplatelet surface, Video S3: Ag diffusion on a PEGylated nanoplatelet surface, Video S4: Cu cluster movement between two lateral PEG chains in a PEGylated graphene surface.
